# Gastric fistula after 20 months of postoperative drain retention: a case report

**DOI:** 10.3389/fsurg.2026.1939915

**Published:** 2026-09-09

**Authors:** Changzhi Chen, Lin Li, Jingfeng Qiu

**Affiliations:** Department of Hepatobiliary and Pancreatic Surgery, The Third Affiliated Hospital of Guangxi Medical University, Nanning, China

**Keywords:** drain-related complication, gastric fistula, intra-abdominal infection, pancreatic fistula, postoperative drain retention, trauma

## Abstract

**Background:**

In trauma or emergency surgery, postoperative drainage may be required for a prolonged period in selected patients because of complex injuries, contamination, or postoperative complications. However, visceral erosion, drain migration, and fistula formation related to long-term drain retention are rare but potentially serious.

**Case presentation:**

In April 2024, a 32-year-old man underwent emergency abdominal surgery after a traffic accident, including laparoscopic cholecystectomy and conversion to open splenectomy because of intraoperative bleeding, and a drainage tube was placed in the splenic fossa. Postoperatively, he developed intra-abdominal infection and postoperative pancreatic fistula. After five weeks of treatment at the outside hospital without significant improvement in symptoms or drainage, he was transferred to our department for further management in June 2024. After admission, computed tomography showed fluid accumulation in the splenectomy bed and an encapsulated collection anterior to the pancreatic body; the original drain was not located within the collection. Percutaneous drainage was performed for the fluid collection anterior to the pancreas. The amylase level in the peripancreatic drainage fluid was 9,526 U/L, compared with a serum amylase level of 75 U/L, supporting the diagnosis of pancreatic fistula. The drain in the splenic region was accidentally dislodged, and a new drainage catheter was promptly inserted through the existing sinus tract. After three weeks of therapy, the patient's symptoms improved and inflammatory markers returned to normal. However, persistent drainage remained, and he was discharged with both drains in place. By September 2024, at the first follow-up visit three months after discharge, the patient still had a daily drainage output of approximately 30–100 mL. The patient was subsequently lost to follow-up. In February 2026, he revisited our department because food residue was observed in the splenic fossa drain. Computed tomography showed that the splenic fossa drain was within the gastric cavity. The peripancreatic drain was removed, and the splenic fossa drain was gradually withdrawn under a clear liquid diet. Three days after complete drain removal, repeat computed tomography showed no intra-abdominal fluid collection and disappearance of the previous sinus tract. At three months after drain removal, the patient remained asymptomatic, with no evidence of recurrent infection or gastric fistula.

**Conclusion:**

Gastric fistula caused by long-term postoperative drain retention is extremely rare. In patients with long-term drainage tubes, the appearance of food residue, gas, abnormal drainage color, or sudden changes in drainage output should raise suspicion of gastrointestinal fistula. Regular follow-up, timely imaging reassessment, and individualized drain removal strategies are essential for preventing delayed drain-related visceral erosion.

## Introduction

Postoperative abdominal drainage is a common surgical practice. Its main purposes include evacuation of blood, fluid collections, leakage from the operative site, and infectious exudates. It also serves as a monitoring tool for early detection of postoperative complications, such as bleeding and anastomotic leakag e (1). In trauma or emergency surgery, postoperative drainage may be required for a longer period in selected patients because of complex injuries, contamination, or postoperative complications. Although long-term drainage may be clinically necessary, the drain itself acts as a foreign body. Prolonged retention may lead to drain obstruction, migration, chronic sinus formation, persistent local infection, and pressure-related erosion of adjacent hollow viscera, eventually resulting in fistula formation (2–5).

Abdominal trauma frequently involves the spleen, and splenectomy remains an effective intervention in selected patients with severe splenic trauma. Postoperative pancreatic fistula (POPF) after splenectomy may occur when the pancreatic tail is injured during trauma surgery, difficult dissection, or management of dense adhesions (6). The diagnosis is supported by elevated amylase levels in drainage fluid. Clinically relevant POPF may require active management, including percutaneous drainage or re-intervention, depending on severity and clinical presentation (7).

Gastric penetration and gastric fistula caused by long-term postoperative drain retention have rarely been reported. Early manifestations may be nonspecific, particularly in patients with pre-existing pancreatic fistula, intra-abdominal infection, and persistent drainage. In such cases, abnormal drainage may be mistakenly attributed to the underlying disease process, leading to delayed diagnosis. Here, we report a rare case of gastric fistula caused by a drainage tube that penetrated the stomach after 20 months of postoperative retention due to intra-abdominal infection and pancreatic fistula. We also review the possible mechanisms, diagnostic considerations, and treatment strategies for this rare complication.

## Case presentation

In April 2024, a 32-year-old man had sustained injuries in a traffic accident and underwent emergency abdominal surgery at another hospital. The surgical procedures included laparoscopic exploration and laparoscopic cholecystectomy, followed by conversion to open splenectomy because of intraoperative bleeding. Postoperatively, the patient developed intra-abdominal infection and POPF. After five weeks of treatment at the outside hospital without significant improvement in symptoms or drainage, he was transferred to our department for further management.

**Figure 1:**
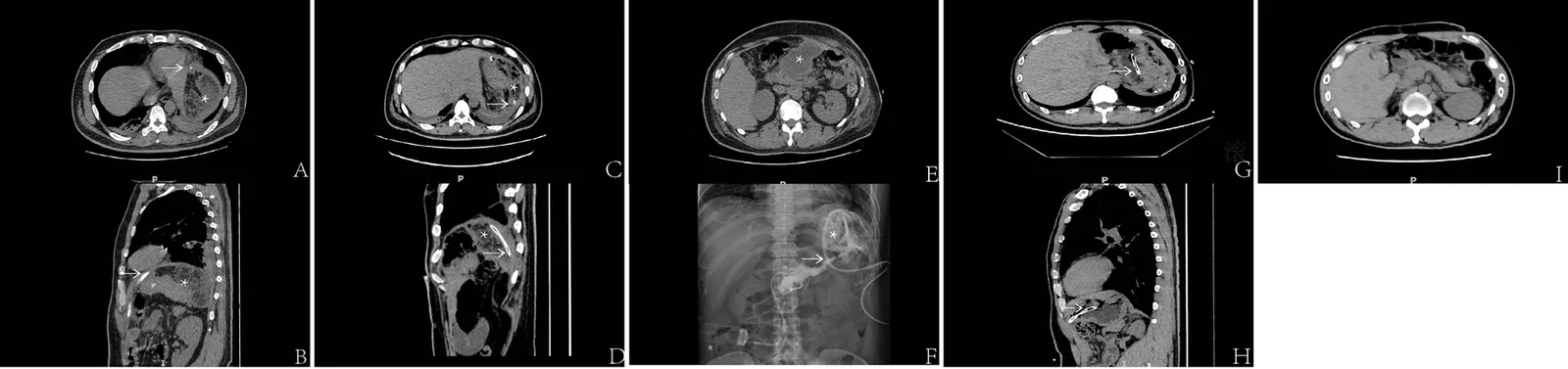
Imaging findings during the clinical course. **(A,B)** Initial CT after transfer showing fluid collection in the splenectomy bed and an encapsulated collection anterior to the pancreatic body; the original drain was not located within the collection. **(C,D)** CT after placement of an 18-Fr silicone tube through the original sinus tract. **(E,F)** Drain tract contrast study showing communication between the peripancreatic drain and the splenic fossa drain. **(G,H)** CT at readmission showing resolution of the previous collections, with the splenic fossa drain visible within the stomach. **(I)** Follow-up CT three days after complete drain removal showing no recurrent intra-abdominal collection. Arrows indicate drainage tubes, and asterisks indicate abscess cavities or encapsulated collections.

On June 2, 2024, upon admission to our hospital, the patient's maximum body temperature was 39.5 °C, and white purulent fluid was observed in the splenic fossa drainage tube. Laboratory findings revealed a white blood cell count of 36.2 × 10⁹/L, neutrophil percentage of 94.2%, platelet count of 553 × 10⁹/L, hemoglobin level of 114 g/L, and procalcitonin level of 2.65 ng/mL. Abdominal computed tomography (CT) demonstrated fluid accumulation in the splenectomy fossa and an encapsulated fluid collection anterior to the pancreatic body. The original drainage tube was malpositioned and was not located within the collection (Figures 1A,B).

On the second day after admission, percutaneous drainage of the collection anterior to the pancreatic body was performed. The drainage fluid was white and purulent. The drainage fluid amylase level was 9,526 U/L, whereas the concurrent serum amylase level was 75 U/L, supporting the diagnosis of pancreatic fistula. Bacterial culture identified Staphylococcus aureus. On the same day, the original splenic fossa drain became dislodged, and an 18-Fr silicone tube was inserted through the original sinus tract. (Figures 1C,D) A drain tract contrast study demonstrated communication between the peripancreatic drainage tube and the splenic fossa drain. (Figures 1E,F).

After three weeks of anti-infective therapy, adequate drainage, and nutritional support, the patient's clinical symptoms improved significantly, and inflammatory markers returned to normal. However, approximately 50 mL of fluid continued to drain daily from the splenic fossa and peripancreatic drains. Because persistent drainage remained, immediate drain removal was considered to carry a risk of recurrent intra-abdominal fluid accumulation and infection. The patient was therefore discharged with the drains in place and was advised to undergo regular follow-up. Three months after discharge, the patient returned for follow-up. Approximately 30–100 mL of drainage fluid was still observed daily from the splenic fossa and peripancreatic drains. The patient did not return for regular follow-up thereafter.

In February 2026, the patient revisited our department because food residue was observed in the splenic fossa drain. Repeat abdominal CT demonstrated resolution of the previous fluid collections anterior to the pancreatic body and in the splenic region. However, the splenic fossa drain was visualized within the gastric cavity (Figures 1G,H). The presence of food residue in the drainage tube, together with CT confirmation of the drain within the gastric cavity, strongly supported the diagnosis of a drain-related gastric fistula.

The patient had no significant abdominal pain, fever, abdominal distension, or other signs of peritonitis, and the intra-abdominal fluid collections had resolved. A 500-mL diluted methylene blue solution was administered orally, however, no blue-stained fluid was observed in the drains, suggesting possible obstruction of the tube. This result was not considered sufficient to exclude a gastric fistula because the distal tip of the drain was later found to be obstructed by food residue.

The peripancreatic drain was removed directly. The splenic fossa drain was then gradually withdrawn under a clear liquid diet: 6 cm on day 1, an additional 2 cm on day 2, and the remaining 3 cm on day 3. Upon final removal, food residue was observed obstructing the distal tip of the drainage tube. This drain-related gastric fistula was classified as Clavien–Dindo grade IIIa because it required interventional management without general anesthesia. Three days after complete removal of all drainage tubes, repeat abdominal CT showed no intra-abdominal fluid collection, and the previous sinus tract had disappeared (Figure 1I). At three months after drain removal, the patient reported no abdominal pain, fever, abdominal distension, nausea, vomiting, or other discomfort. There was no evidence of recurrent intra-abdominal infection or gastric fistula. The complete clinical course is summarized in Table 1.

**Table 1:** Clinical timeline of the patient.

Date	Clinical event
25 Apr 2024	Road traffic accident and emergency surgery at an outside hospital; hospitalized at the referring hospital for approximately 5 weeks.
2 Jun 2024	Transferred to our hospital.
4 Jun 2024	Percutaneous drainage was performed for the collection anterior to the pancreas; The original splenic fossa drain was accidentally dislodged and an 18-Fr silicone tube was reinserted through the original sinus tract.
24 Jun 2024	Discharged with both drains in place after approximately 3 weeks of hospitalization at our hospital; persistent drainage remained.
25 Sep 2024	First post-discharge follow-up; drainage volume was approximately 30–100 mL/day.
6 Feb 2026	Food residue was observed in the splenic fossa drain, and CT showed that the drain had penetrated into the gastric cavity.
8–10 Feb 2026	The peripancreatic drain was removed, followed by progressive withdrawal and complete removal of the splenic fossa drain over 3 days.
13 Feb 2026	Follow-up CT showed no recurrent intra-abdominal fluid collection.
8 May 2026	Three-month follow-up after drain removal; the patient remained asymptomatic, with no recurrent infection or gastric fistula.

## Discussion

This report describes an extremely delayed gastric fistula caused by a postoperative drainage tube retained for approximately 20 months. Unlike most previously reported drain-related gastrointestinal injuries, which occurred within the first postoperative week or within one month after surgery, the present case developed after a prolonged period of drain retention (8–10). This unusually delayed presentation makes the case clinically important and highlights the need for long-term surveillance in patients discharged with drainage tubes.

Several factors may have contributed to gastric fistula formation in this case. First, the initial operation was performed in an emergency trauma setting. Compared with elective surgery, emergency trauma surgery is often associated with limited preoperative preparation, complex intra-abdominal injuries, tissue edema, contamination, bleeding, and a higher risk of postoperative infection or leakage. These factors may increase the need for prolonged drainage and make early drain removal more difficult. Second, prolonged drain retention was the most direct cause. Continuous contact between the drain and the gastric wall may have resulted in local pressure, ischemia, chronic inflammation, and eventual erosion. Third, postoperative pancreatic fistula and intra-abdominal infection may have aggravated local inflammation. Pancreatic juice and infectious exudates can promote adhesion formation and sinus tract development, keeping the gastric wall in close contact with the drain and surrounding inflamed tissues. Fourth, after dislodgement of the original drain, an 18-Fr silicone tube was inserted through the original sinus tract. This tube was relatively firm and may have increased local pressure on adjacent gastric tissue. Finally, irregular follow-up after discharge prevented timely imaging reassessment of the drain position and delayed drain removal or replacement.

Management of peripancreatic fluid collections includes a range of approaches, such as endoscopic ultrasound-guided transgastric drainage, percutaneous drainage, laparoscopic drainage, and open surgical drainage. The choice of treatment should be individualized according to the patient's clinical condition, the location and characteristics of the collection, and the presence of infection or necrosis. In our patient, percutaneous drainage was initially selected because the collection anterior to the pancreas was localized and accessible, and there was no indication for immediate surgical exploration.

The treatment strategy was selected according to the patient's clinical stability and imaging findings. There was no obvious peritonitis, the previous encapsulated intra-abdominal collections had resolved, and long-term drain retention suggested that the sinus tract had become relatively mature. In addition, obstruction of the drain tip by food residue suggested that active leakage might have been limited. Therefore, we chose gradual withdrawal of the drain rather than surgical or endoscopic intervention. On the first day of withdrawal, the drain was retracted by 6 cm, corresponding to the estimated length of the catheter located within the gastric cavity. Follow-up computed tomography after drain removal showed no intra-abdominal fluid collection and disappearance of the sinus tract, indicating that this conservative strategy was successful in the present patient.

This case provides several important clinical lessons. First, postoperative drains should not be retained indefinitely. Even when persistent drainage is present, the source of drainage, drain position, and feasibility of removal should be reassessed periodically. Second, patients discharged with drains must undergo regular follow-up, especially those with pancreatic fistula, bile leakage, or intra-abdominal infection. Third, the material, stiffness, diameter, and position of a drain may influence the risk of delayed pressure-related erosion. For patients who require long-term drainage, a soft tube of appropriate diameter should be selected, and its position should be regularly confirmed. Fourth, the appearance of food residue, gas, bile-like fluid, feculent material, or sudden changes in drainage output should immediately raise suspicion of gastrointestinal fistula.

This report has several limitations. First, gastroscopy or upper gastrointestinal contrast study was not performed; therefore, the gastric fistula opening was not directly visualized. Second, because the patient was lost to follow-up, the exact time at which the drainage tube penetrated the stomach could not be determined. Third, this is a single case report, and whether conservative treatment with gradual tube withdrawal is applicable to other similar patients requires accumulation of additional cases and further validation.

## Conclusion

Long-term retention of a postoperative abdominal drain may cause rare but serious delayed visceral erosion and fistula formation. Food residue in a drainage tube is an important warning sign of gastrointestinal fistula. In selected patients without peritonitis and with resolved intra-abdominal collections, gradual drain withdrawal under a clear liquid diet may be a feasible conservative treatment option.

## Data Availability

The original contributions presented in the study are included in the article/Supplementary Material, further inquiries can be directed to the corresponding author.
